# Plasmonic oligomers in cylindrical vector light beams

**DOI:** 10.3762/bjnano.4.6

**Published:** 2013-01-24

**Authors:** Mario Hentschel, Jens Dorfmüller, Harald Giessen, Sebastian Jäger, Andreas M Kern, Kai Braun, Dai Zhang, Alfred J Meixner

**Affiliations:** 1 4th Physics Institute and Research Center SCoPE, University of Stuttgart, Pfaffenwaldring 57, 70569 Stuttgart, Germany; 2 Max Planck Institute for Solid State Research, Heisenbergstrasse 1, 70569 Stuttgart, Germany; 3 Institute of Physical and Theoretical Chemistry, University of Tübingen, Auf der Morgenstelle 18, 72076 Tübingen, Germany

**Keywords:** near-field microscopy, oligomers, plasmons, radial and azimuthal polarization

## Abstract

We investigate the excitation as well as propagation of magnetic modes in plasmonic nanostructures. Such structures are particularly suited for excitation with cylindrical vector beams. We study magneto-inductive coupling between adjacent nanostructures. We utilize high-resolution lithographic techniques for the preparation of complex nanostructures consisting of gold as well as aluminium. These structures are subsequently characterized by linear optical spectroscopy. The well characterized and designed structures are afterwards studied in depth by exciting them with radial and azimuthally polarized light and simultaneously measuring their plasmonic near-field behavior. Additionally, we attempt to model and simulate our results, a project which has, to the best of our knowledge, not been attempted so far.

## Introduction

Plasmonics is the optics of metal nanoparticles. If an external light field impinges on a metal nanoparticle, collective oscillations of the quasi-free conduction electrons are excited. The electron charge cloud is displaced with respect to the fixed ionic background and thus causes local electric fields. The main benefit afforded by plasmonics is its ability to concentrate incoming electromagnetic energy into deep subwavelength volumes, so-called hot spots. The local electric-field strength can surpass the incoming field strength by orders of magnitude. The process is moreover surprisingly efficient as the plasmonic resonances couple extremely well to an external light field due to the huge resonant dipole moment, which is fundamentally connected to the large number of free conduction electrons.

Just as atoms join together in order to form molecules, plasmonic particles can couple to one another and form collective states. In molecular physics this coupling is mediated via the electron wavefunctions, which mix and hybridize giving rise to new collective orbitals [1]. In plasmonics this coupling is mediated by the plasmonic near fields. In contrast to molecular physics, plasmonics allows us to nearly arbitrarily change the spatial arrangement, the number, and the properties of the constituent particles [2–3]. We can thus tailor the light–matter interaction at will and create novel optical components and devices [4–8].

We investigated the excitation as well as propagation of magnetic modes in such plasmonic nanostructures. We studied the magneto-inductive coupling between adjacent nanostructures and derived the necessary prerequisites for the efficient launch of magnetic plasmon propagation. In our experiments we utilized high-resolution electron-beam lithography for the fabrication of the nanostructures. In order to study the excitation of magnetic modes we used a home-built combined near-field scanning and confocal microscope. The structures were excited with azimuthally and radially polarized light, which allows for an efficient excitation of the fundamental magnetic modes. All these concepts and devices are going to be introduced and discussed in detail in the following.

## Results and Discussion

### Azimuthal and radial fields: theoretical description

**Figure 1:**
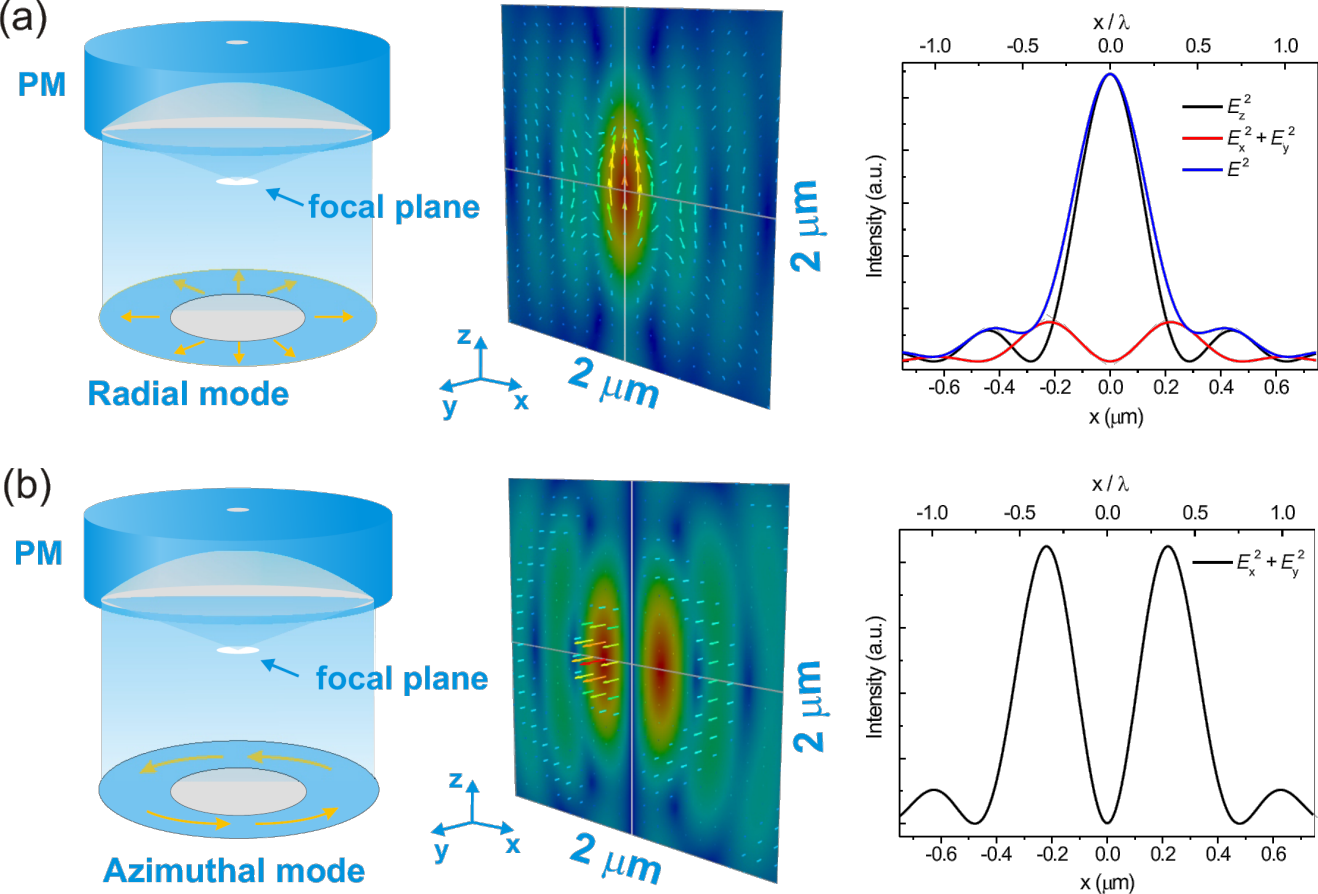
(a) and (b) show sketches of simulated focus patterns (*E*^2^ in *xz* plane) in the parabolic mirror and their intensity profile. Adapted from [11].

Azimuthal and radial laser modes, also known as cylindrical vector beams, create unique field distributions when focused by high numerical aperture (NA) parabolic mirrors or objective lenses, as depicted in Figure 1. In such a high-NA focus of an azimuthally polarised beam, only in-plane polarized fields are present. By focusing the radially polarized laser beam, a distinctively different field distribution is present. Here a strong *z*-polarized field is present in the center surrounded by a weak in-plane polarized field. The ratio between the *z*- and in-plane polarized field is directly correlated with the NA [9–10].

The calculation of the interaction of radially and azimuthally polarized light with plasmonic nanostructures is a surprisingly complex problem. The mainly utilized methods, such as FDTD, are particularly suited and optimized for plane-wave excitation. Radial and azimuthal fields, however, require a special basis set as they can no longer be described with a single *k*-vector but show a strong variation in direction and magnitude of the *k*-vector contributions in the tight focus of the incoming radiation. Simulation techniques such as Fourier-modal methods, however, are covered by extended fields with discrete *k*-vectors. Thus, the problem cannot be implemented by using these simulation techniques. The huge computational power that would be required to solve the resulting multiscale problems is simply unavailable.

Starting from this need, we developed two sets of techniques in order to tackle the problem at hand. One possibility relies on the so-called multiple-multipole method (MMP). It is a semi-analytical simulation theory based on Mie scattering. The simulated field distribution is described by a sum of distributed expansions, which are analytical solutions of Maxwell’s equations, and the coefficients of the expansions are solved at the boundaries. In particular, the technique takes advantage of the high symmetry of the clusters, which significantly eases the required computational power. Once the correct expansion has been found, the field distributions as well as the far-field scattering spectra can be calculated from the analytical solutions of Mie theory.

Another description has been developed by using the surface integral equation (SIE) method. This approach uses Green's functions to describe the propagation of an electromagnetic field in homogeneous media and can thus reduce the computational domain to material interfaces. Enforcing the boundary conditions at the material boundaries, the surface fields can be computed for arbitrary incident conditions. Given these surface fields, the field distribution in the surrounding space can then be derived.

A big advantage of the SIE method is the use of continuous basis functions to describe the surface fields. This leads to a physical solution of a boundary value problem at the interfaces, yielding accurate field distributions even in the extreme near field of the particles. Due to the definition of the Green's functions up to infinite distances, the field distribution is accurately reproduced in the far field as well. In addition, as the space surrounding the particles does not need to be discretized, implementation of arbitrary incident conditions, such as cylindrical vector beams, is a simple task.

### Plasmonic oligomers

Originally, we intended to utilize split-ring resonators (SRR) as magnetic atoms. These U-shaped nanostructures support a plasmonic mode which is associated with a strong magnetic moment. Arranging SRRs in a chain and thus coupling these modes would allow for a magnetic-plasmon propagation and hence for efficient and low-loss energy propagation on the nanoscale. Yet, the experimental realization of such structures that exhibit a resonant response in the required wavelength regime around 630 nm proved to be too challenging.

**Figure 2:**
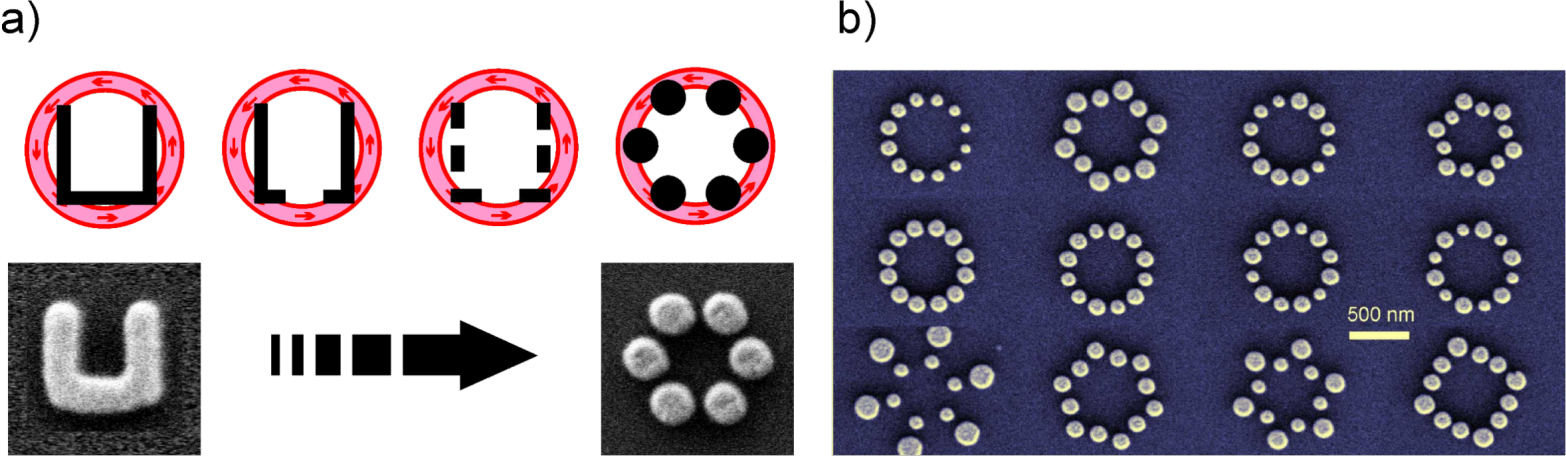
(a) Replacing the split ring resonators with individual plasmonically coupled gold dots allows us to spectrally shift the supported modes while retaining most of the optical properties. (b) Additionally, ring-like arrangements of gold nanoparticles are particularly suited for experiments with radially and azimuthally polarized light as they are perfectly matched to their high spatial symmetry.

We thus developed an alternative route, which proved to be highly fruitful, cf. Figure 2. By replacing the closed metal-rings of the SRRs by individual but strongly coupled metal nanoparticles, we were able to retain a number of the described properties of the SRRs and yet drastically blue-shift the resonant optical response of the resulting clusters. The ring-shaped arrangements of the gold nanoparticles are ideally matched to the radially and azimuthally polarized excitation and form collective plasmonic modes exhibiting electric as well as magnetic nature. Moreover, these clusters support a multitude of *tunable collective modes*, which justify studying these kinds of plasmonic artificial molecules themselves in detail, even under linearly polarized light [12–16].

**Figure 3:**
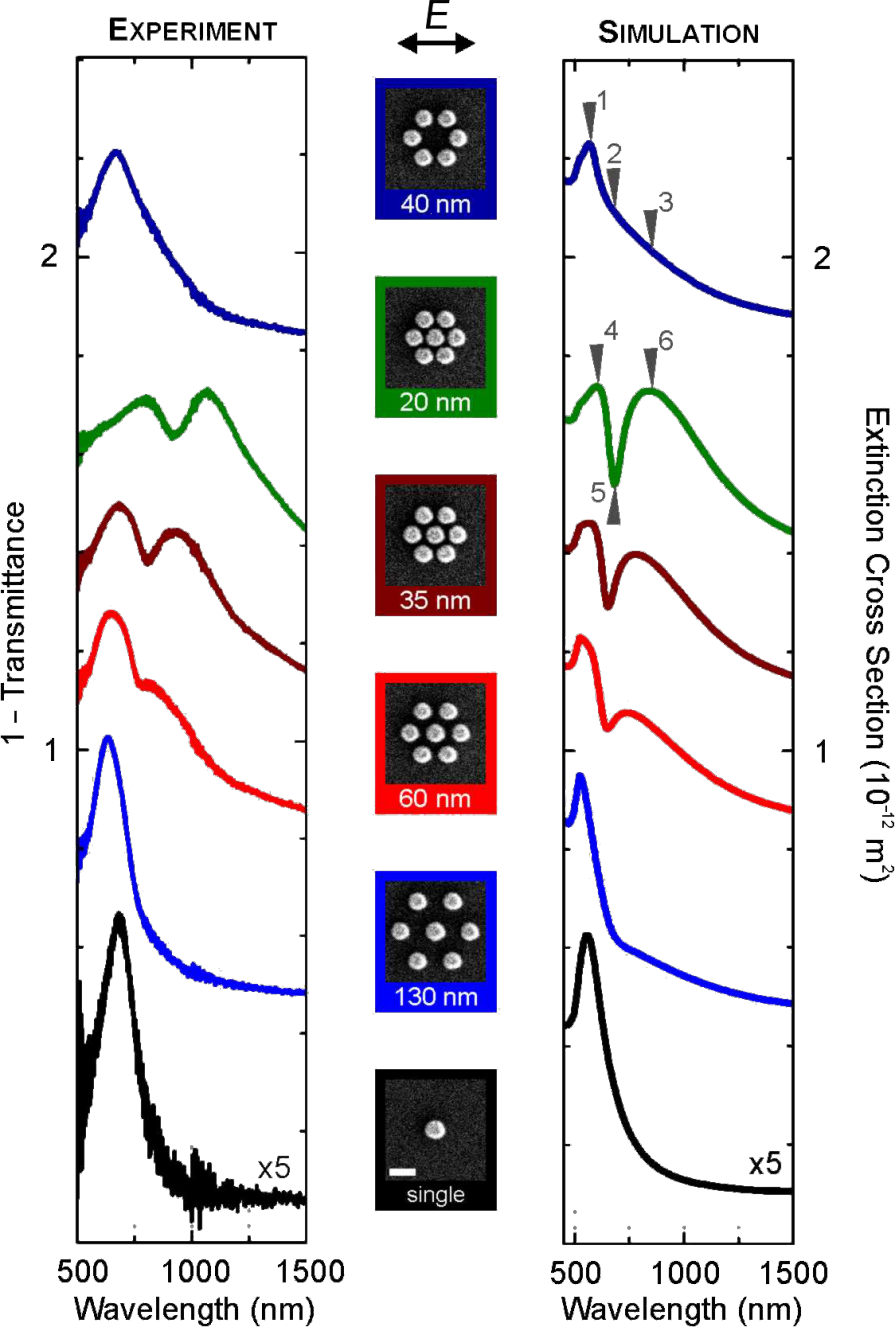
Extinction spectra of a gold monomer, a gold hexamer, and gold heptamers with different interparticle gap separations. The spectra are shifted upward for clarity. (left column) Experimental extinction spectra (1 − transmittance). (middle column) SEM images of the corresponding samples with indicated interparticle gap distances. The scale-bar dimension is 500 nm. (right column) Simulated extinction cross-section spectra by using the multiple-multipole method. Adapted with permission from [13]. Copyright (2010) American Chemical Society.

In the following we briefly review earlier work on plasmonic oligomers [13]. In order to investigate the evolution of the coupling behavior in plasmonic oligomers, we studied the optical response of a series of nanoparticle oligomers with various interparticle gap distances, cf. Figure 3. The interparticle gap distance *g* was decreased from 130 to 20 nm. For excitation of the structures, we used normal incident light with linear polarization, as shown in Figure 3, left column. The experimental spectra of the samples and their corresponding SEM images are displayed in the same figure. The spectrum of the gold monomer is plotted as a black curve in the bottom row. A single dipolar resonance is observed around 700 nm (the curve is magnified by a factor of 5 for better comparability). Turning toward the heptamer with a large interparticle gap distance (*g* = 130 nm), the spectrum shows approximately the same behavior as the isolated nanoparticle due to the well-separated nanoparticle configuration. This would correspond in molecular chemistry to the situation of uncoupled atoms before they start to form molecular bonds. As the interparticle gap distance is reduced (*g* = 60 nm), a second peak starts to form around 800 nm. The two peaks are separated by a pronounced dip. As the interparticle gap distance is further reduced towards *g* = 20 nm, the spectral features red-shift successively. In the ring-like hexamer (*g* = 40 nm), which we display for comparison (see the top black spectrum), the shorter-wavelength peak around 700 nm is also present. In contrast to the heptamers, no pronounced dip is visible in the hexamer. We rather observe a long and unstructured tail toward the long-wavelength region.

Figure 3 as well presents the simulated extinction spectra for different structures. The spectra have been calculated by using the MMP method [17]. It is apparent that the experimental results show a good qualitative agreement with the numerical predictions. The overall red shift of the experimental spectra with respect to the simulated spectra is due to the presence of the glass substrate in the experiment. The difference between the experimental and simulated results is also partially due to the assumption of a nanosphere shape for the trapezoidal nanoparticles in the simulation. Nevertheless, all the main spectral features including the distinct resonance dip are clearly predicted.

**Figure 4:**
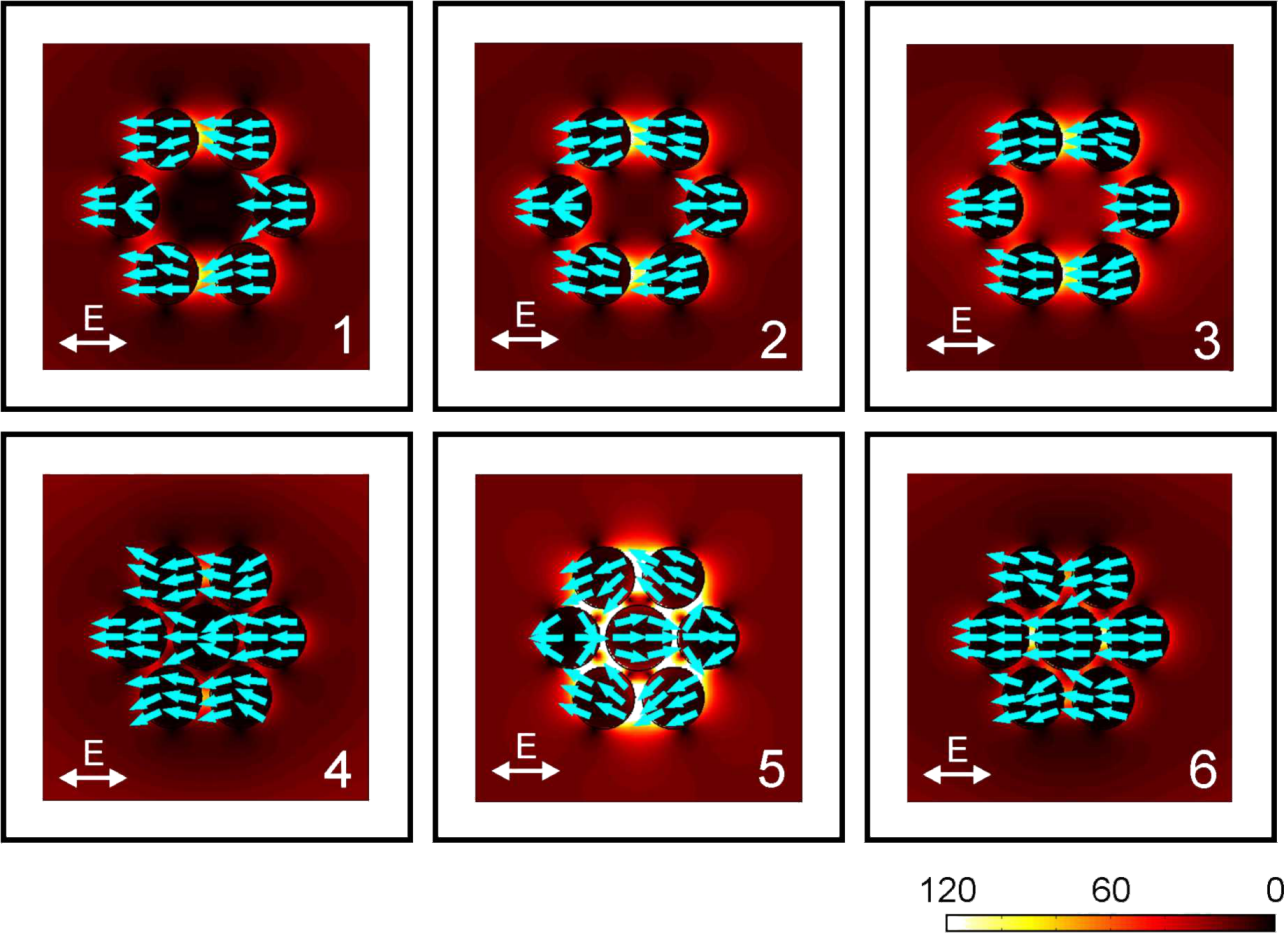
Simulated field distributions and local electric currents (blue arrows) for the gold hexamer and heptamer at the respective spectral positions (as indicated by the arrows in the simulated spectra) by using the multiple-multipole method. The color scale indicates the absolute value of the electric field relative to the external exciting field strength. It is notable that at spectral position 5 in the heptamer, similar yet opposite oscillating plasmons are excited in the central nanoparticle and the ringlike hexamer, thus leading to a subradiant mode. The destructive interference between the subradiant mode and the broad super-radiant mode results in the Fano resonance. In the absence of the central nanoparticle, the nanoparticles in the hexamer always oscillate in phase, leading to a collective dipolar mode. Adapted with permission from [13]. Copyright (2010) American Chemical Society.

In order to elucidate the character of the resonances, field distributions at the respective spectral positions are shown in Figure 4. In the hexamer structure, at spectral positions 1, 2, and 3, the currents in the six nanoparticles always oscillate in-phase, manifesting the excitation of the collective dipolar plasmon resonance in the ringlike hexamer. In the heptamer structure, when a central nanoparticle is brought into close proximity with the six satellite nanoparticles, the dipolar plasmon of the central nanoparticle hybridizes with the hexamer dipolar plasmon, giving rise to the formation of a bright super-radiant collective mode and a dark subradiant collective mode. For the super-radiant mode, the oscillating plasmons in the seven nanoparticles are in-phase (see field distributions at spectral positions 1 and 3), exhibiting significant mode broadening due to radiative damping.

It is worth mentioning that the peak position of the super-radiant mode cannot be exactly determined from the spectrum due to the presence of the resonance dip. Nevertheless, the resonant behavior at positions 1 and 3 is a good indication for the super-radiant mode. For the subradiant mode, the net sum of the plasmon polarizations of the six satellite nanoparticles oscillates oppositely with respect to the plasmon polarization in the central nanoparticle (see field distribution at spectral position 2). The unique symmetry of the heptamer allows for similar yet opposite dipole moments of the central nanoparticle and the ringlike hexamer, thus leading to a narrow mode. The formation of the distinct dip in the spectrum is due to the destructive interference between the narrow subradiant mode and the broad super-radiant mode, which is called a Fano resonance [18–20].

**Figure 5:**
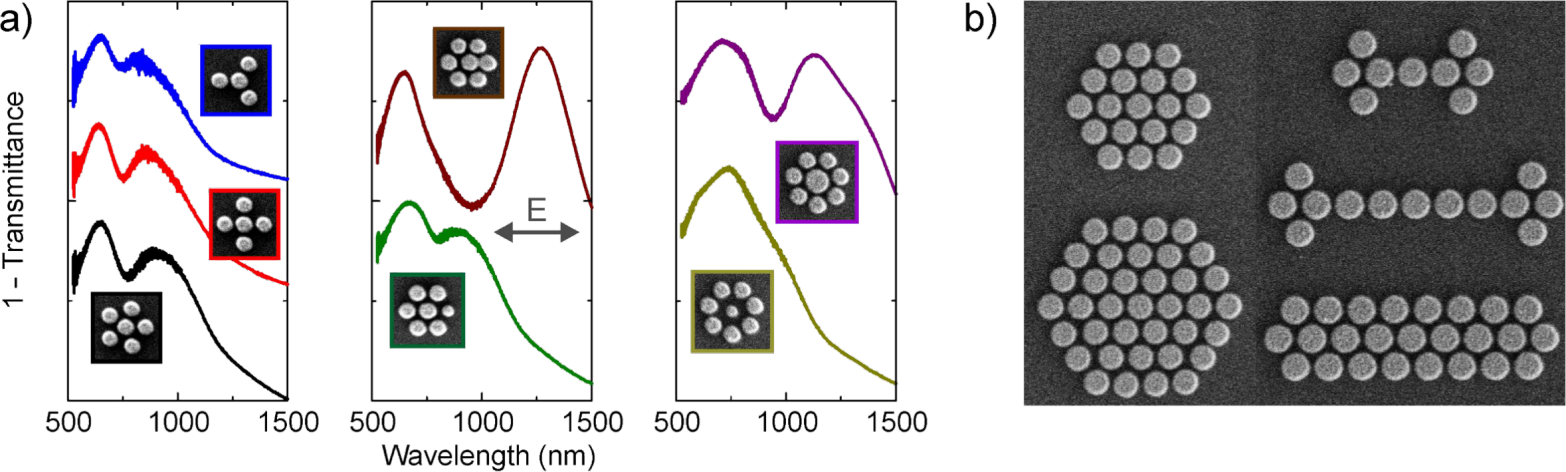
(a) Examples of complex plasmonic oligomers with tailorable optical properties. Adapted with permission from [21]. Copyright (2011) American Chemical Society. (b) Scanning-electron micrographs of oligomers clusters, demonstrating the unique capability of electron-beam lithography to create many of the different spatial arrangements imaginable.

The oligomeric design strategy is highly tunable and allows us to nearly arbitrarily manipulate the optical spectra. Figure 5a depicts SEM micrographs and spectra of exemplary oligomers. Changing the number, size, and spatial arrangement of the individual particles, allows for the tuning of the strength and spectral position of the transparency window. Under certain conditions, the Fano resonance even vanishes completely [21].

Electron-beam lithography is a highly controllable top-down technique that can be utilized in order to fabricate the structures. Nearly every manipulation in the design of the cluster can be easily implemented. Figure 5b depicts a collection of SEM micrographs that demonstrate our ability to create nearly every arrangement imaginable [22–26].

### The optical near-field microscope

**Figure 6:**
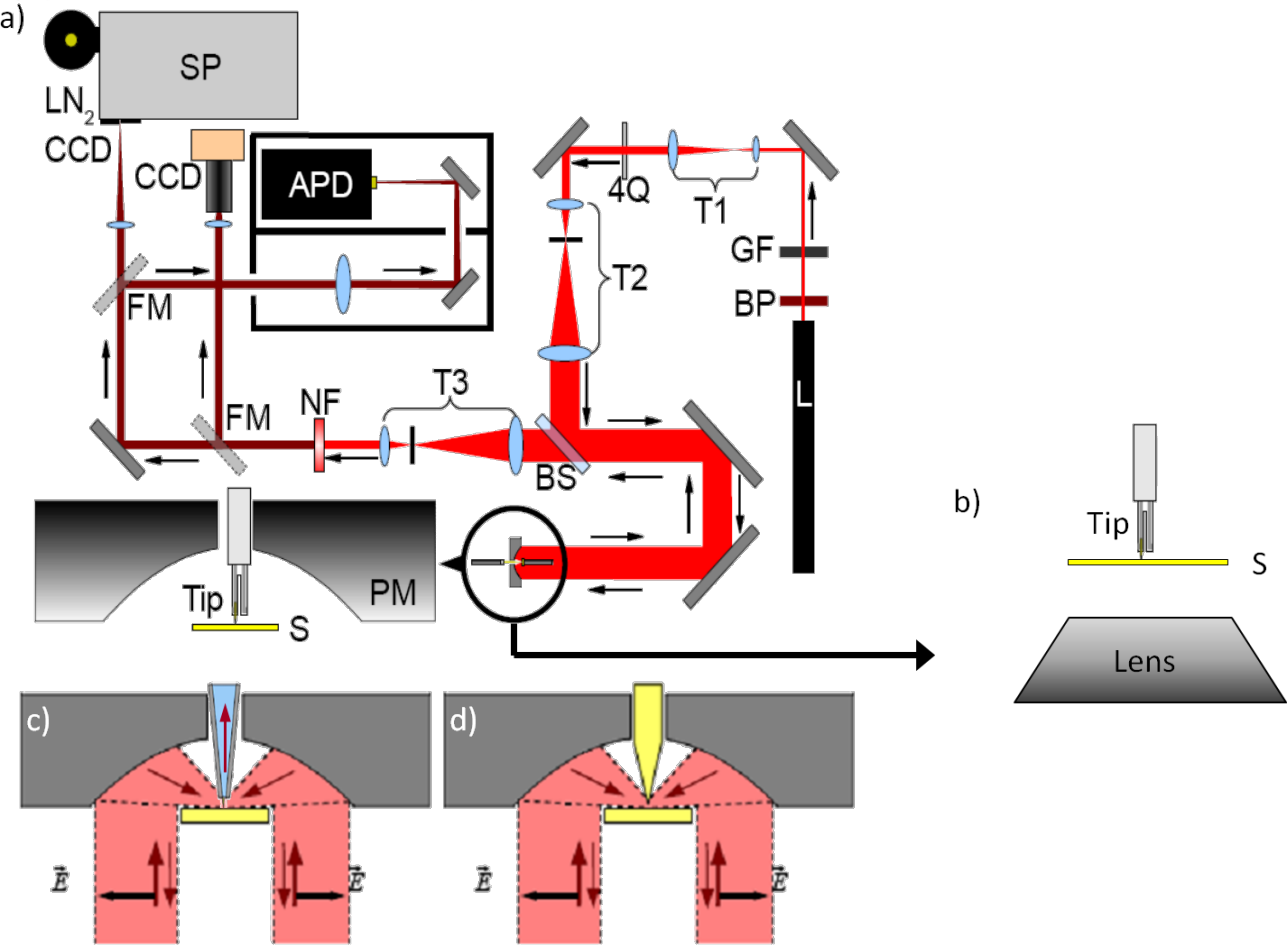
Schematic of two optical microscopic configurations using a parabolic mirror for focusing (a, adapted from [27]) and an objective lens for focusing (b). Shear-force SNOM configurations can be obtained by integrating a glass fiber (c) or a gold tip (d) to the confocal optical microscopes.

The studies of the optical behavior of the plasmonic oligomers with focused radially and azimuthally polarized laser beams were performed on home-built combined near-field scanning and confocal microscopes. The exciting laser is in both cases a 632.8 nm HeNe laser, where the beam is transformed by a mode conversion into radially or azimuthally polarized cylindrical vector beams. One of the microscopes uses an oil immersion objective lens (NA of 1.25) for focusing. The other microscope uses a parabolic mirror with an NA of 0.998 as the focusing element (Figure 6). Based on these confocal microscopes, in combination with a home-built shear force AFM system and the use of sharp gold or glass-fiber tips, we operate a versatile scanning near-field optical microscope (SNOM) system.

To measure the near field generated from the plasmonic oligomers, one single oligomer is placed inside the radially or azimuthally polarized focus. A sharp gold tip (tip radius smaller than 15 nm) is used to scan over the structure, collecting the near-field responses. Notably, the sharp gold tip also gives a strong photoluminescence signal, whose intensity is linearly proportional to the local field strength at the plasmonic structures.

### Experiments and simulation of near-field imaging of plasmonic oligomer rings using gold luminescence

We excited the plasmonic oligomer rings with radially and azimuthally polarized light at 632.8 nm. The excitation was performed by utilizing a parabolic mirror with an NA = 0.998. The detection was performed by confocal microscopy and the collected signal is one-photon photoluminescence.

**Figure 7:**
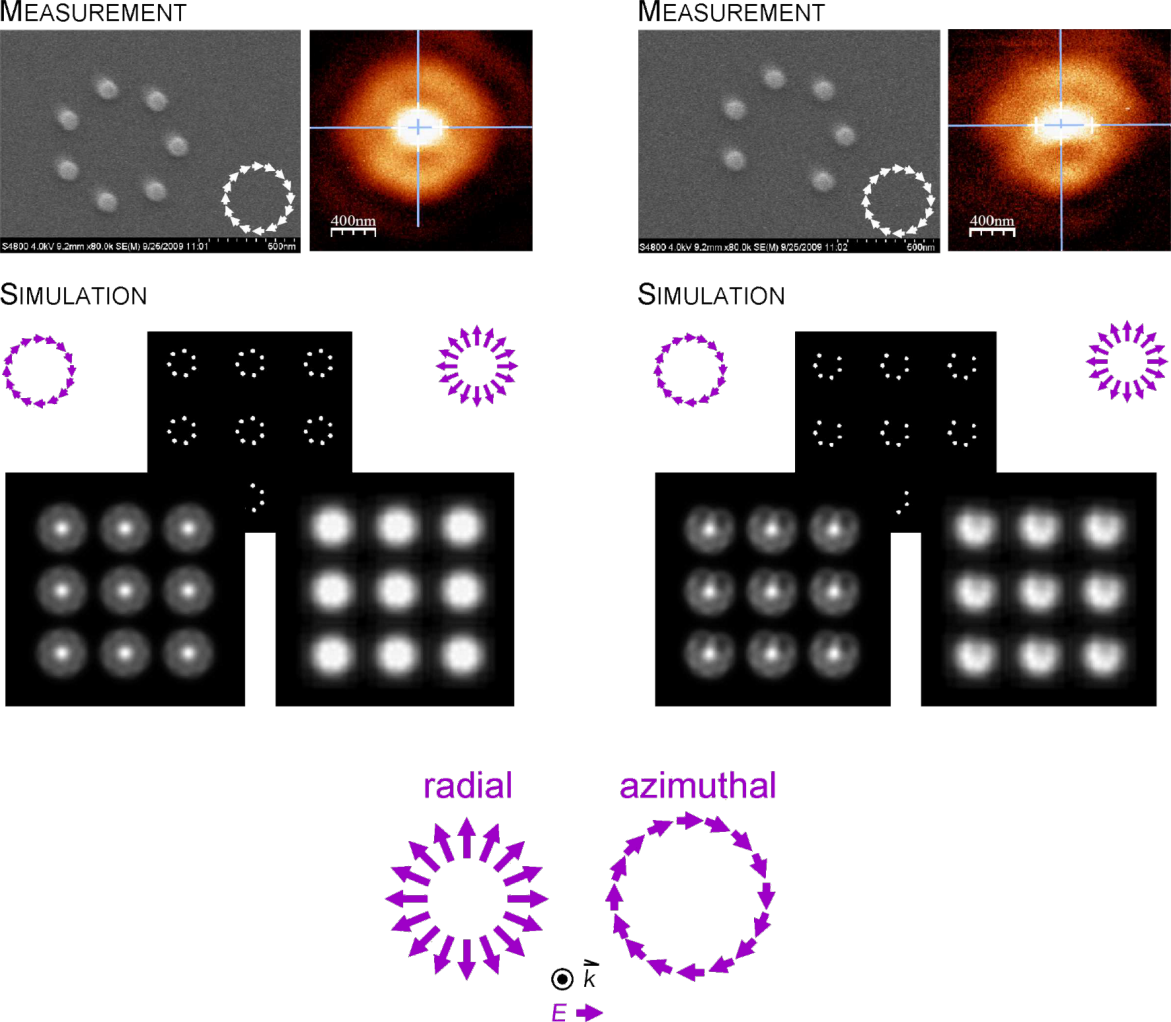
Oligomer rings composed of gold nanodots, SEM images on the left, confocal luminescence images under azimuthally polarized illumination on the right. Lower row: Simulations based on a planar convolution of the exciting light field with the structure geometry. The left image depicts the one-photon photoluminescence image upon azimuthal excitation. This geometry corresponds to the measured configuration shown in the upper row. The right image shows the predicted signal upon radial excitation. The lowest row depicts once more a simplified sketch of the used polarization states.

Figure 7 depicts examples of these kinds of measurements on closed and open oligomer ring structures. One observes a strong interaction of the light field with the plasmonic nanostructures, resembling a “magnetic focusing”, featuring a central spot of enhanced intensity with a diameter of ca. 340 nm. The reason for this behavior is as follows: The luminescence signal is generated by the individual gold dots, which in turn have been excited by the external light filed. Thus, the signal strength is given by the superposition of the excitation spot and its intensity distribution with the geometry of the oligomer rings. A convolution of these two quantities describes the observed phenomenon.

### Near-field microscopy of aluminium heptamers

**Figure 8:**
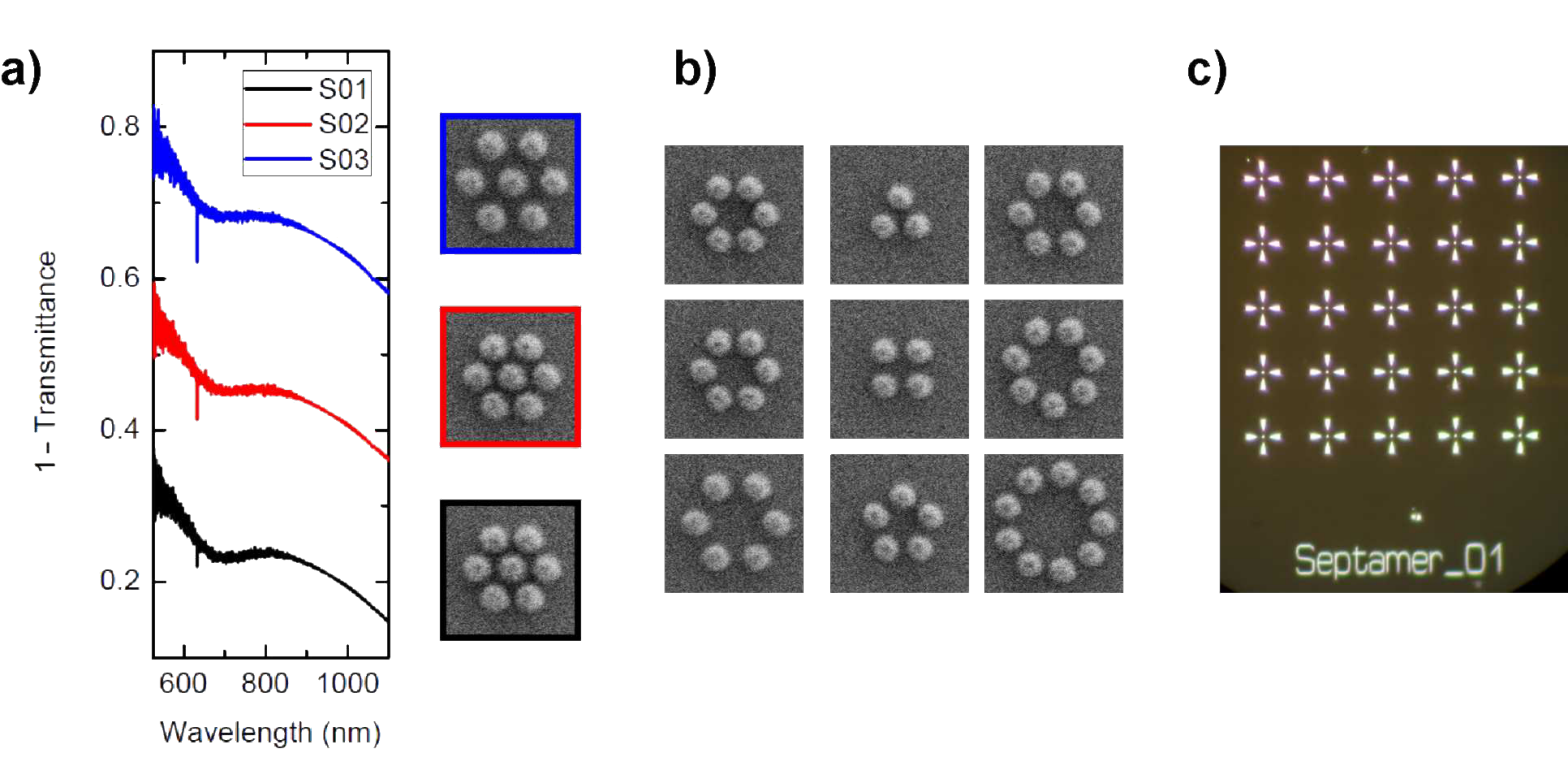
Oligomer structures consisting of aluminium. (a) The formation of collective behavior in the clusters is visible by the formation of a well-pronounced Fano resonance. (b) Varying the number of ring particles allows us to study the evolution of the spectra. Starting from a certain number of ring particles one expects the spectral evolution to saturate, similarly to the transition from a molecule to a solid. (c) The individual clusters are surrounded by marker structures, allowing easy location of the structures on the substrate during measurement.

During the progress of our experiments we found that the gold structures show substantial photoluminescence [28–30]. For some of the conducted experiments, as discussed above, this phenomenon is beneficial, yet, it turned out to be mostly bothersome. In order to circumvent this problem, we designed oligomer structures consisting of aluminium. The new material affords two benefits: on the one hand the photoluminescence of aluminium is significantly smaller than the one of gold. On the other hand the significantly higher plasma frequency of aluminium causes the plasmonic resonances to blue-shift for identical particle sizes, easing fabrication. Figure 8 depicts spectra, SEM, and optical micrographs of these structures.

### Simulation of the near-fields of plasmonic oligomers under radially and azimuthally polarized excitation

**Figure 9:**
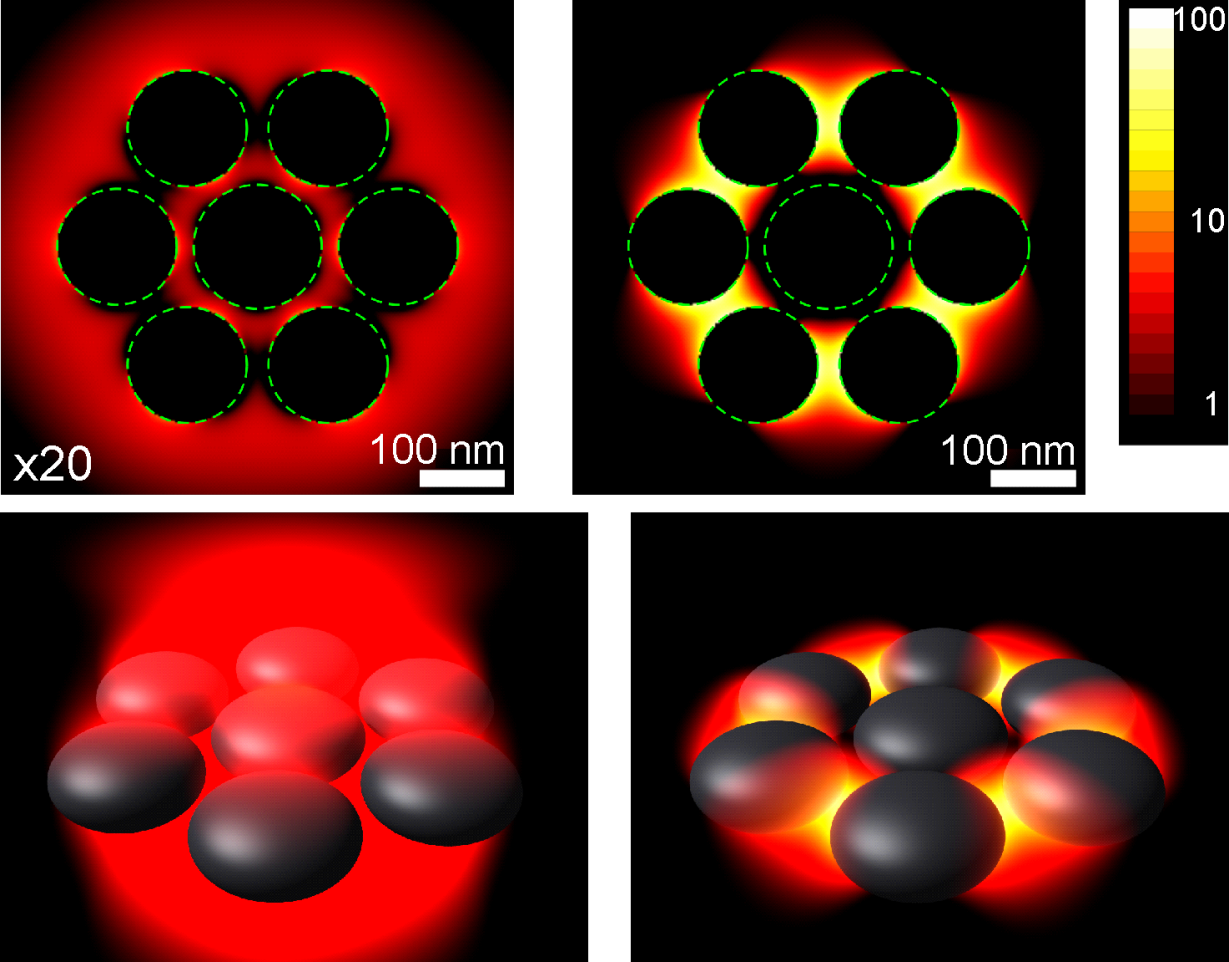
Simulated near-fields of heptamer structures under radial (left, multiplied by 20 for a better comparability) and azimuthal (right) excitation. The upper row depicts the field strength in the center plan, whereas the lower row depicts the same data as a 3-D plot. One can clearly observe the strong enhancement of the electric fields between, within the nanoscale gaps. The overall field strength is significantly higher for azimuthal excitation as this perfectly fits the symmetry of the cluster and the arrangement of the gaps.

Utilizing the above-discussed surface-integral method [31–33] we were able to calculate the electric near-field distributions of a plasmonic oligomer under radial and azimuthal excitation. The upper row of Figure 9 depicts the near-field intensity distribution within the symmetry plane of the cluster; the lower row shows a 3-D plot of the same data. In both excitation geometries we observe a strong near-field enhancement resembling the high symmetry of the cluster itself. The strong enhancement, as well as the strong local fields, is proof that we indeed excite the cluster with its eigenpolarizations. Such near-field patterns, in strength as well as spatial distribution, are not obtainable upon linear polarized excitation. The field enhancement for azimuthal excitation is significantly stronger as it perfectly matches the nanoscale gaps at the circumference of the cluster. The field is mostly concentrated within these gaps, whereas nearly no field localization is associated with the center particle. This behavior is in striking contrast to the case of linear-polarized excitation (cf. Figure 2, right column). In the case of radial excitation the near fields are mostly confined to the gap between the outer particles and the center one. Overall, the field distribution again resembles the cluster symmetry.

### Magnetic plasmon propagation along oligomer chains

Oligomers support magnetic modes. Hence, it is a straightforward idea to combine these kinds of structures to form chains, which then in turn allow for the coupling between these modes and thus the transport of energy along the chains. As the coupling will be mediated by the coupling between magnetic dipoles, the transport is expected to show low loss [34–35]. However, measuring such magnetic plasmon propagation requires local excitation at one of its ends and the near-field distributions to be measured along the whole chain [36–38]. This measurement technique requires the separation of the excitation and collection foci, which is experimentally extremely challenging.

## Conclusion

We presented a route to create magnetic excitations and propagation on the nanoscale by utilizing individual yet strongly near-field coupled plasmonic atoms. These atoms join together and form collective plasmonic modes which are associated with strong magnetic moments. Arranging these plasmonic molecules in a chain and thus coupling these modes allows for magnetic plasmon propagation and, hence, for efficient and low-loss energy propagation on the nanoscale.

We have demonstrated our ability to create plasmonic molecules with tailorable optical properties. We studied these molecules using far- and near-field measurement techniques. In particular, we observed the transition from isolated to collective modes upon decreasing the interparticle gap. We were able to directly measure the near-field distributions of our plasmonic molecules with cylindrical vector light beams, which, to the best of our knowledge, had not been demonstrated before.

Moreover, we have developed simulation techniques in order to model our results on these complex structures, which constitute a significant advance in the understanding of the interaction of our unconventional states of polarization with the complex plasmonic structures.
